# The IgE-diagnostic impact of a recombinant serine protease inhibitor from *Hevea brasiliensis* in latex-allergic health care workers

**DOI:** 10.1186/2045-7022-4-S2-P56

**Published:** 2014-03-17

**Authors:** Hans-Peter Rihs, Ingrid Sander, Heike Heimann, Ursula Meurer, Thomas Brüning, Monika Raulf-Heimsoth

**Affiliations:** 1 Institute for Prevention and Occupational Medicine of the German Social Accident Insurance (IPA), Molecular Genetics, Bochum, Germany; 2 Institute for Prevention and Occupational Medicine of the German Social Accident Insurance (IPA), Allergology/Immunology, Bochum, Germany; 3 Institute for Prevention and Occupational Medicine of the German Social Accident Insurance (IPA), Head of the Institute, Bochum, Germany

## Background

Serine protease inhibitors (SPI) comprising 60-90 amino acid residues, are frequent in plants, and belong to the protein family PR-6. It has recently been shown that a SPI-variant of wheat acts as an important allergen in baker's asthma but not in persons suffering from wheat-induced food allergy. The aim of this study was to elucidate the role of a recombinant SPI-variant of *Hevea brasiliensis* for occupational latex allergy.

## Methods

For this purpose a cDNA from *Hevea brasiliensis* was used to amplify the SPI-specific sequence. After identification by sequencing and transfer into the pMAL-system, the sequence was expressed as maltose-binding protein (MBP)-SPI hybrid in *E.coli*. The soluble purified fusion protein was biotinylated and coupled to Streptavidin-ImmunoCAP. Sera of 21 health care workers (HCW) with allergic symptoms to natural rubber latex (NRL) were tested.

## Results

Specific IgE (sIgE)-values >0.35 kUA/L were regarded as positive. Seven sera (33%) displayed SPI-sIgE (range: 0.56-13.60 kUA/L). Out of these seven sera all displayed sIgE to rHev b 6.01 (range: 1.81- 66.80 kUA/L) and with one exception also sIgE to rHev b 5 (1.86-49.6 kUA/L). Furthermore, positive sIgE to nHev b 2 (n=6/7) and rHev b 7 (n=4/7) were most frequently. No sIgE to MBP was detectable.

## Conclusions

The results show that this SPI-variant is a further latex allergen, which is available now for extended tests, for instance on microarray platforms. The proposed name Hev b 15 is currently under review of the WHO/IUIS allergen nomenclature subcommittee. Especially in patients showing discrepancies between the sum of sIgE-values to single NRL-allergens and the IgE-value to NRL k82, Hev b 15 may be one candidate filling the gap.

